# Highly Sensitive Bacteriophage-Based Detection of *Brucella abortus* in Mixed Culture and Spiked Blood

**DOI:** 10.3390/v9060144

**Published:** 2017-06-10

**Authors:** Kirill V. Sergueev, Andrey A. Filippov, Mikeljon P. Nikolich

**Affiliations:** Department of Bacteriophage Therapeutics, Bacterial Diseases Branch, Walter Reed Army Institute of Research, Silver Spring, MD 20910, USA; kirill.v.sergueev.ctr@mail.mil (K.V.S.); andrey.a.filippov.ctr@mail.mil (A.A.F.)

**Keywords:** *Brucella abortus*, brucellosis, phage-based detection, phage amplification, qPCR, rapid assay, blood and clinical samples

## Abstract

For decades, bacteriophages (phages) have been used for *Brucella* species identification in the diagnosis and epidemiology of brucellosis. Traditional *Brucella* phage typing is a multi-day procedure including the isolation of a pure culture, a step that can take up to three weeks. In this study, we focused on the use of brucellaphages for sensitive detection of the pathogen in clinical and other complex samples, and developed an indirect method of *Brucella* detection using real-time quantitative PCR monitoring of brucellaphage DNA amplification via replication on live *Brucella* cells. This assay allowed the detection of single bacteria (down to 1 colony-forming unit per milliliter) within 72 h without DNA extraction and purification steps. The technique was equally efficient with *Brucella abortus* pure culture and with mixed cultures of *B*. *abortus* and α-proteobacterial near neighbors that can be misidentified as *Brucella* spp., *Ochrobactrum anthropi* and *Afipia felis*. The addition of a simple short sample preparation step enabled the indirect phage-based detection of *B*. *abortus* in spiked blood, with the same high sensitivity. This indirect phage-based detection assay enables the rapid and sensitive detection of live *B*. *abortus* in mixed cultures and in blood samples, and can potentially be applied for detection in other clinical samples and other complex sample types.

## 1. Introduction

Brucellosis is a global bacterial zoonotic disease of high social and economic importance. It is debilitating and disabling for hundreds of thousands of people and causes high reproductive losses in food animals every year [1,2]. The causative agents of livestock and human brucellosis are small Gram-negative facultative intracellular coccobacilli in the genus *Brucella*, which are slow growing and typically form visible colonies after 48–72 h of incubation on agar media. Six classic terrestrial and two marine pathogenic species of *Brucella* have been identified, though the discovery of a number of new species over the last decade has continued to expand our understanding of the genus. Four of the classic terrestrial *Brucella* species can cause human brucellosis: *Brucella melitensis* (primary hosts: goats and sheep), *Brucella abortus* (cattle), *Brucella suis* (swine), and *Brucella canis* (dogs). The marine mammal pathogen species *Brucella ceti* and *Brucella pinnipedialis* have also been reported to infect humans [3,4]. During the Cold War, *Brucella* spp. were weaponized [5], and nowadays the U.S. Centers for Diseases Control (CDC) still categorizes *B*. *melitensis*, *B*. *abortus* and *B*. *suis* as Category B Select Agents [6].

Since the symptoms of human brucellosis are nonspecific, clinical diagnosis is problematic and must be confirmed by laboratory tests. Laboratory diagnosis is also challenging, especially early in infection [7,8]. The CDC outlines the following three laboratory criteria for definitive diagnosis of human brucellosis: (i) isolation of *Brucella* spp. from the blood or reticuloendothelial tissue biopsies (bone marrow); (ii) fourfold or greater rise in *Brucella* agglutination titer between acute- and convalescent-phase serum specimens obtained two weeks or more apart and studied at the same laboratory; and (iii) demonstration by immunofluorescence of *Brucella* spp. in a clinical specimen [9]. Every criterion is sufficient for confirmation of the disease, but isolation and phenotypic characterization of *Brucella* spp. is still considered to be the gold standard and ultimate proof of brucellosis [7].

Despite the use of selective media to improve the isolation and identification of *Brucella* [10,11,12,13], and the successful use of automated culture systems [8,14,15], *Brucella* isolation remains challenging. The process is laborious (often including sample preparation), lengthy (usually taking 3–45 days of cultivation and additional time for subsequent phenotypic identification), associated with risk of laboratory-acquired infections, and has a relatively low sensitivity: 0–90%, depending on the phase of infection, the least efficient in chronic cases [7,8,14,16].

Unlike bacterial culture, serological testing is rapid, less hazardous, less expensive, and more sensitive; and is thus used preferentially in clinical practice. However, these methodologies also have several disadvantages: (a) a positive serologic assay (detection of *Brucella* total antibody titer of ≥1:160) is only considered presumptive unless there is a fourfold or greater titer rise; (b) serologic tests are often negative at early stages of infection; (c) diagnostic titers do not necessarily correlate with active infection; (d) assays should be standardized for different animal species and human populations, depending on the levels of endemicity; (e) there are numerous reports of false-positive serologic tests caused by cross-reactivity to epitopes in O-polysaccharide shared between *Brucella* and *Yersinia enterocolitica* O:9, *Salmonella urbana*, *Vibrio cholerae*, *Francisella tularensis*, *Escherichia coli* O:157 and *Stenotrophomonas maltophilia*. In addition, the use of smooth live attenuated *Brucella* vaccine strains in animals complicates serologic differentiation between natural and vaccine-induced infection because of the presence of lipopolysaccharide O antigen [7,8].

Detection of *Brucella* DNA in clinical samples using various PCR assays has proved to be rapid (down to few hours), efficient at different stages of the disease, more specific than serological tests, and more sensitive than blood cultures. The use of different DNA targets provides identification at the level of genus (which is sufficient to start antibacterial therapy) or at the level of species (which is important for epizootological and epidemiological analysis). Real-time PCR allows for high-throughput screening of clinical specimens and improves analytical sensitivity. Multiplex PCR assays enable genotyping and strain characterization [8,17,18,19,20,21]. However, the generally low concentrations of *Brucella* and the presence of PCR inhibitors in blood and tissues still complicate the molecular diagnosis of brucellosis, and thus PCR tests require sample preparation, including bacterial DNA extraction, concentration, and purification. The DNA diagnostic criteria for active infection are yet to be established, because there are many cases when *Brucella* DNA is repeatedly detected for months or even years after antibacterial therapy without recovery of live bacteria and in the absence of any symptoms of brucellosis. The interpretation of these results is unclear because PCR assays cannot distinguish between live and dead, or culturable and unculturable bacteria [8,20,22,23,24].

Routine bacteriophage lysis tests have been used for diagnosis of brucellosis for more than 60 years [25]. Brucellaphages Tbilisi (Tb), Firenze (Fz), Weybridge (Wb), S708, Berkeley (Bk), R/C, and Izatnagar (Iz) have very similar morphology and genome structure, but differ in their specificity [26,27,28,29,30]. The use of this set of phages at two different concentrations allows the definitive identification of *Brucella* at both genus and species level, including differentiation between smooth and rough strains of *B*. *abortus*, *B*. *suis*, *B*. *melitensis*, *B*. *neotomae*, *B*. *canis*, and *B*. *ovis* [26,31,32,33]. Phage-based identification is a multi-day assay, including the isolation of a pure *Brucella* culture, and a plaque assay using the double-layer agar technique, requiring 48–72 h of incubation [26,32].

We have recently proposed a rapid (4–6 h) and simple indirect bacteriophage-based approach for the detection and identification of *Yersinia pestis*, the causative agent of plague [34]. The test was based on monitoring amplification of *Y*. *pestis*-specific phages in the presence of the live bacteria using real-time quantitative PCR (qPCR). The detection limit was as low as a single bacterium per 1-µL sample (10^3^ colony-forming units (CFU)/mL) and could even be reduced by sample concentration. The method worked well on simulated clinical blood, serum and tissue samples, and did not require DNA isolation [34,35]. This technology was demonstrated to have the same efficiency whether using a laboratory bench-top qPCR machine or a portable instrument designated for field work [35].

The purpose of this study was to develop a qPCR assay targeting brucellaphages—such as Tb, S708 or Bk—for indirect identification of *B*. *abortus* and related *Brucella* spp. Phage propagation was delayed, but robust, and enabled the detection of single *B*. *abortus* cells per milliliter of sample within 72 h. Higher concentrations of bacteria resulted in shortened turnaround time (24–48 h). The method also allowed reliable detection of single *B*. *abortus* cells in simulated blood samples within 72 h and identification of *Brucella* in mixed cultures containing relative α-proteobacteria *Ochrobactrum anthropi* or *Afipia felis*.

## 2. Results

### 2.1. Brucellaphage Propagation and DNA Extraction

Diagnostic brucellaphages Tb, S708, Fz, Wb, and Bk were propagated to high titers (Table 1). It was determined that the use of conical Erlenmeyer flasks with vented closures and shaking at 60 rpm are critical for efficient phage propagation. Flasks were filled to no more than 1/5 of their nominal volume to avoid spillage and to assure the adequate aeration. The bacteriophage titer of each new lysate was determined, and a portion of each lysate was used for phage DNA extraction and purification. Table 1 presents phage concentration in infectious particles and phage genome equivalents per milliliter. The latter was determined using the calibration curve established by qPCR (see the next section).

### 2.2. Performance Testing of qPCR with Phage DNA and Intact Phage Particles

**Figure 1:**
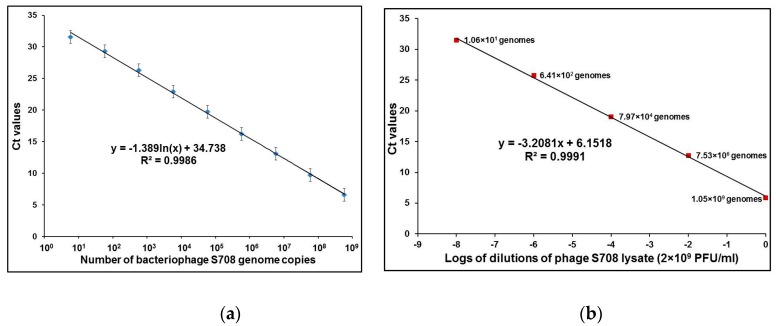
qPCR of purified DNA of brucellaphage S708 (**a**) and S708 lysate (**b**). Calibration curve (**a**) was established using qPCR of 1 µL aliquots of nine different 10-fold dilutions of purified DNA from brucellaphage S708, ranging from 0.35 fg to 3.5 ng. Calibration curve (**b**) was established using qPCR of 1 µL aliquots of several 100-fold dilutions of phage S708 lysate, ranging from ~10^0^ to ~10^8^ PFU. The number of viable particles per sample was confirmed by plaque assays Ct: cycle threshold.

The quantitative parameters of bacteriophage-based qPCR detection were first tested by using serial 10-fold dilutions of purified DNA from brucellaphage S708. These experiments were done in three separate runs, each in duplicate, and yielded a log linear function between DNA concentrations and threshold cycle number (Ct), spanning a 9-log dilution series, from 3.5 ng down to 0.35 fg of DNA (Figure 1a). We calculated that this corresponds to about 10 to 1.0 × 10^9^ S708 genome equivalents, based on the fact that its genome size is 38,253 bp [31]. To compare qPCR results obtained using purified DNA and intact phage particles, a series of 100-fold dilutions of phage S708 lysate was prepared in saline magnesium (SM) buffer, and qPCR reactions were run with different concentrations of phage particles ranging from 10^0^ to 10^8^ PFU per 1 µL of phage lysate (per 10 µL of qPCR sample). The number of viable particles per sample was confirmed by plaque assays. The results of enumeration of phage particles by qPCR (Figure 1b) were one to two logs higher in comparison with plaque counts, and in case of phage Bk this difference was up to four logs (see also Table 1). This could be explained by partial inactivation of brucellaphages with chloroform [36], and the results may suggest that Bk is the phage most sensitive to chloroform. However, since qPCR of brucellaphage S708 DNA indicated that the actual number of phage genomes per sample is at least 10 times greater than the number obtained by plaque count, the assay sensitivity was not compromised. Very similar results were obtained with genomic DNA of other brucellaphages (data not shown).

### 2.3. Sensitivity of Phage-Based qPCR Detection of *Brucella abortus* in Broth Culture

**Figure 2:**
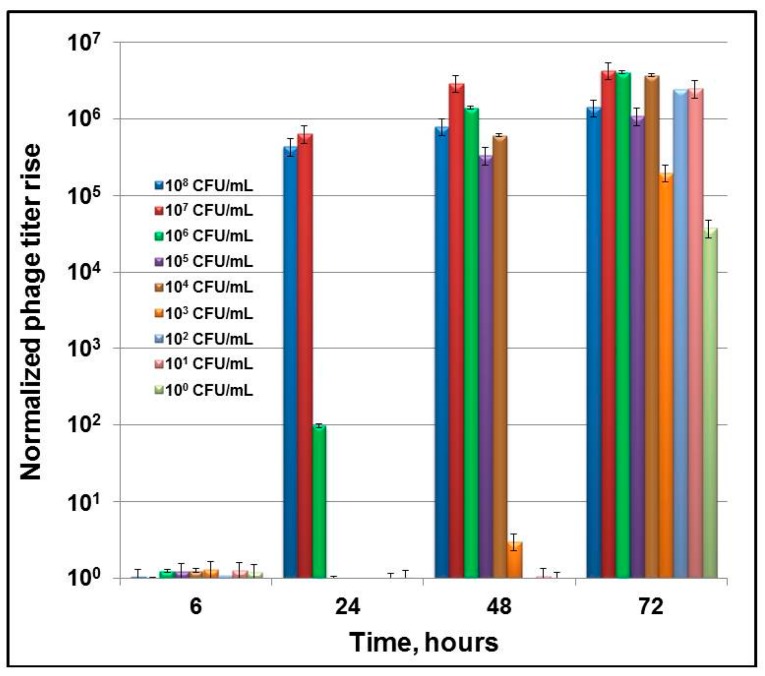
Dynamics of phage S708 growth on *B*. *abortus* S19 detected by qPCR. An overnight culture of *B*. *abortus* S19 was diluted to OD_600_ = 0.1 in Brucella Broth. 20 mL of each serial bacterial dilution from 10^0^ to 10^−8^ (circa 10^8^ to 10^0^ CFU/mL, respectively) was made in Brucella Broth. Diagnostic bacteriophage S708 was added to each aliquot to a final concentration of 10^3^ PFU/mL, and the sample was incubated in a shaker incubator at 37 °C and 60 rpm. 500 µL samples were withdrawn after 0, 6, 24, 48, and 72 h of incubation, treated with 10% chloroform and centrifuged to remove cell debris. 1 µL of each sample, neat or diluted 1:100 with Brucella Broth, was used for qPCR analysis. Reaction mixes were prepared in a total volume of 10 µL including 5 µL of the master mix and 0.3 µM of each primer. The cycling parameters were: 95 °C, 5 min; 40× (95 °C, 20 s; 60 °C, 60 s) with fluorescence measurement at the end of each cycle.

**Figure 3:**
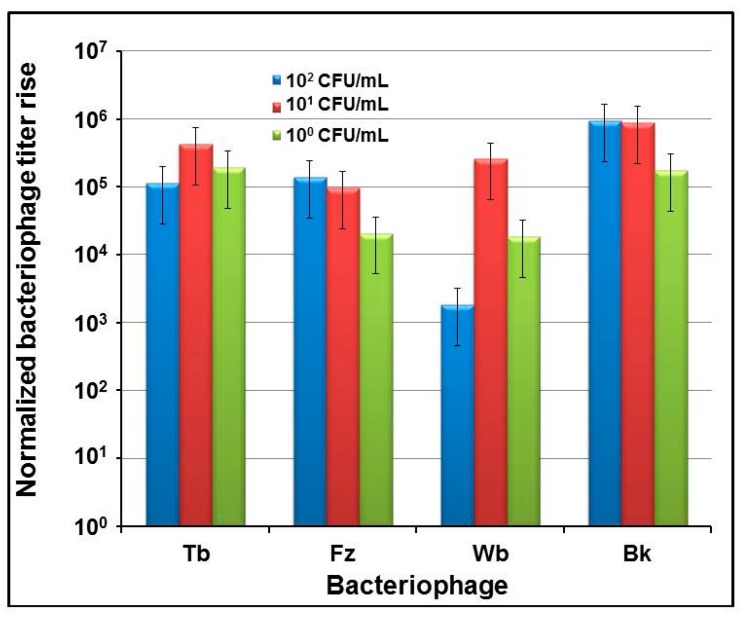
Amplification of brucellaphages Tb, Fz, Wb, and Bk on low concentrations of *B*. *abortus* S19. Phage lysates were diluted in saline magnesium (SM) buffer and added at 10^3^ PFU/mL to the dilutions of bacterial culture in Brucella Broth at concentrations of ~1 × 10^2^; ~1 × 10^1^ and ~1 × 10^0^ CFU/mL, which were confirmed by live bacterial counts. qPCR was performed after 72 h of incubation of each test culture with the corresponding phage.

To define the sensitivity of indirect phage-mediated detection of *B*. *abortus*, first a series of experiments was conducted in which diagnostic brucellaphage S708 was propagated on *B*. *abortus* cultures at different concentrations ranging from 1 × 10^8^ to about 1 CFU/mL. The use of several starting concentrations of the S708 phage showed that 10^3^ PFU/mL is the minimum titer, providing clear and consistent phage amplification at any bacterial concentration (data not shown). This starting phage titer was then used in all further experiments. The increase in phage titer was detected by qPCR (Figure 2), applying Ct values to the calibration curve (Figure 1a). The lowest amount of phage providing reliable positive qPCR signal was determined to be 100 phage genome equivalents per 20 µL sample (containing 1 µL of phage lysate). This number was normalized to 1 (Figure 2). Our data showed that the detection of *B*. *abortus* is robust and there are clear differences in phage yields at different concentrations of *B*. *abortus* over the course of time. We were unable to detect any rise in phage titer over 6–8 h of incubation. The first detectable phage burst was observed after 24 h of incubation at higher initial concentrations of *B*. *abortus* ranging from 10^8^ to 10^6^ CFU/mL. This increase in phage concentration was unexpectedly large: over five orders of magnitude. After 48 h of incubation, a similar 5 times log burst was noted at lower *B*. *abortus* concentrations (10^5^–10^3^ CFU/mL). Finally, a 72 h incubation resulted in the robust phage titer increase at the lowest concentrations of *B*. *abortus* (10^2^–10^0^ CFU/mL). Thus, the sensitivity of S708-mediated qPCR assay was ~10^0^ CFU/mL. The lower limits of detection were also determined for several other brucellaphages: Tb, Fz, Wb, and Bk. The results are presented in Figure 3. Each phage from the diagnostic panel produced a robust burst detected by qPCR. The highest titer rise was observed with the Tb and Bk phages. The vigorous amplification of Bk that enabled efficient indirect detection of *B*. *abortus* was of particular interest, since this phage can propagate on a number of *Brucella* spp. [32,37,38], and thus its use in this approach should increase the spectrum of detected *Brucella* spp. Based on this finding, we used the Bk phage in most further experiments.

### 2.4. Specificity of qPCR Tests with the Bk Bacteriophage

**Figure 4:**
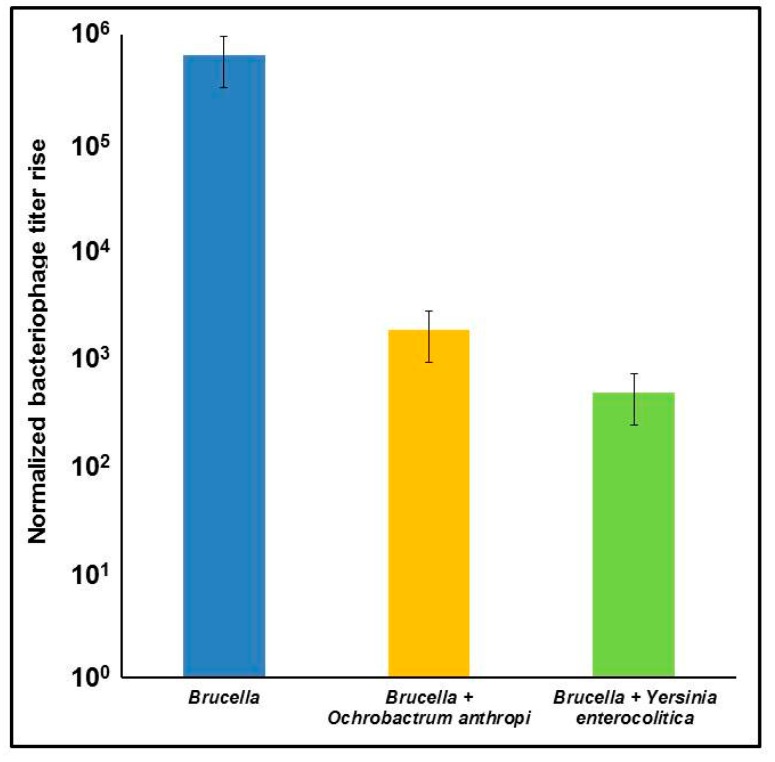
Brucellaphage Bk propagation on mixed bacterial cultures. Bacterial cultures overnight in Brucella Broth. The cultures were diluted 1:1000 with Brucella Broth and brucellaphage Bk was added to get a final concentration of 10^3^ PFU/mL and approximate multiplicity of infection (MOI) of 1:1000. All cultures were incubated at 37 °C for 72 h with shaking at 200 rpm, treated with 10% chloroform; then a 0.5-mL aliquot was taken and centrifuged to remove cell debris. One microliter of each lysate was used for the qPCR reaction. For testing phage specificity in mixed cultures, equal volumes of diluted overnight non-*Brucella* and *B*. *abortus* S19 cultures were mixed, grown in the presence of the Bk phage and processed as described above.

Specificity of the phage-based detection method was tested on six bacterial species belonging to different taxonomic groups, including two representatives of α-proteobacteria close to *Brucella*, *O*. *anthropi* and *A*. *felis*, as well as *Y*. *enterocolitica* serovar O:9 carrying antigens cross-reactive with *Brucella* (see Table 2). We did not observe any propagation of the Bk phage after 72 h incubation with any of the tested bacterial cultures except *B*. *abortus* (data not shown), suggesting that the assay is specific. Moreover, the Bk phage was shown to robustly amplify on *B*. *abortus* in the mixed cultures with faster-growing *O*. *anthropi* or *Y*. *enterocolitica* O:9 (Figure 4), though some degree of inhibition of the phage propagation in comparison with the control *Brucella* culture was observed, especially in the presence of *Y*. *enterocolitica* O:9. However, the phage titer rise was still remarkably high, and easily detectable by qPCR.

### 2.5. Assay Performance with Simulated Clinical Samples

To test if the qPCR phage-based assay can be used for the detection of *Brucella* spp. in blood, we used different commercially available sheep blood products including whole blood with different anticoagulants such as sodium citrate, ethylenediaminetetraacetic acid (EDTA), heparin, and acid citrate dextrose (ACD), as well as defibrinated blood, plasma and serum (all purchased from LAMPIRE Biological Laboratories, Inc., Pipersville, PA, USA). Blood cells were removed from whole and defibrinated blood by centrifugation, and early-logarithmic-phase culture of *B*. *abortus* S19 was added to the resulting serum or plasma to a final concentration of 10^7^ CFU/mL. The mix was diluted 1:10 with Brucella Broth to provide the better growth conditions for *Brucella*, and brucellaphage S708 was added at 10^3^ PFU/mL. After 72 h of incubation at 37 °C, we were unable to detect any rise in the phage titer by qPCR, regardless of the final dilution of the sample. Similar results were observed when the S708 phage was added to commercially available sheep plasma or serum and when using other brucellaphages with different sheep blood products (data not shown). Since a 5 times log increase in brucellaphage titers was observed before in Brucella Broth, the negative result with all blood products suggested that serum component(s) may inhibit brucellaphage propagation.

**Figure 5:**
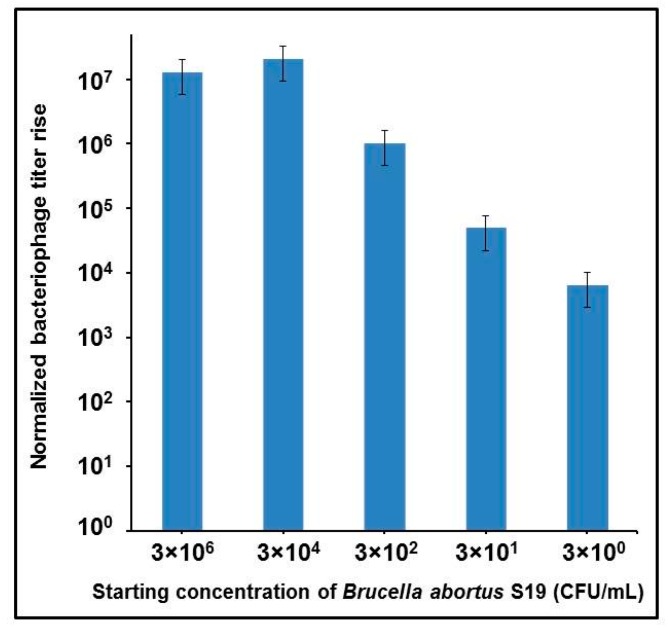
Amplification of the Bk phage after 72 h growth on different concentrations of *B*. *abortus* S19 in simulated infected blood samples. The tests were performed in Na_2_- ethylenediaminetetraacetic acid (EDTA)-treated whole sheep blood. An overnight culture of *B*. *abortus* S19 was diluted to OD_600_ = 0.1 in Brucella Broth. Serial bacterial dilutions 10^−2^ (~3 × 10^6^ CFU/mL), 10^−4^ (~3 × 10^4^ CFU/mL), 10^−6^ (~3 × 10^2^ CFU/mL), 10^−7^ (~3 × 10^1^ CFU/mL), and 10^−8^ (~3 × 10^0^ CFU/mL) were made in sheep blood. To avoid inhibition of phage infection by components of sheep serum, the samples were processed using a lysis-centrifugation procedure (see Section 4.6). Determination of live brucellae was performed by plating 100 µL of 10^−6^ dilution of the washed blood sample. The entire processed blood sample was inoculated into 20 mL of Brucella Broth. Phage was added at 10^3^ PFU/mL, and the samples were incubated at 37 °C with shaking at 60 rpm for 72 h, then treated with 10% chloroform and centrifuged to remove cell debris. One microliter of each sample was used for qPCR analysis.

In order to address this inhibition, a simple and short (about 10 min) sample preparation step was added that included removal of erythrocytes, lysis of leukocytes and washing of the bacterial cells (see Materials and Methods). Sheep blood with Na_2_-EDTA was spiked with *B*. *abortus* at different concentrations, and the loss of bacteria over centrifugation steps was monitored. We observed not more than a 20% decrease of *Brucella* live count in the final water suspension in comparison with the initial blood sample. Bacteriophage Bk was selected for this test because of its broad host range [32,37,38]. After 72 h of incubation, robust phage amplification was revealed by qPCR even at the highest bacterial dilution, corresponding to single *Brucella* cells per milliliter of blood (Figure 5).

## 3. Discussion

Laboratory diagnosis of brucellosis is a challenging and often time-consuming task. The best proof of the infection is isolation of *Brucella* culture from clinical samples followed by standard bacteriological characterization. Culturing *Brucella* poses among the highest risks of laboratory-acquired aerosol infection, while the isolation rate of these fastidious bacteria from blood is relatively low, and subsequent phenotypic bacteriological identification is time-consuming. Thus, current laboratory diagnosis is mainly based on serologic and molecular methods, but none of these approaches can be used on its own for consistent and reliable diagnosis. For example, serologic tests are ineffective early in the infection, sometimes have limited specificity because of cross-reactive antibodies and, in multiple observations, no correlation was found between antibody titers and clinical picture, including seronegative brucellosis patients and seropositive tests after effective antibiotic treatment. Many PCR assays targeting *Brucella* DNA have been developed, but these cannot discriminate between live and dead bacteria, and there are still no established DNA diagnostic criteria for active brucellosis, since *Brucella* DNA can be detected long after antibiotic treatment in the absence of culture of live bacteria or any brucellosis symptoms. Thus, the question of whether a positive DNA test in clinical samples should be followed by prolonged antibacterial therapy is often debatable [7,8,24]. In addition, despite the plethora of sensitive and specific PCR assays that have been developed against *Brucella* pathogens, the detection of infection in clinical specimens using PCR is still not widely accepted to be reliable, and we have experience with multiple DNA extraction techniques and highly sensitive real-time PCR assays failing to detect *Brucella* in human blood samples from which *Brucella* spp. were cultured [39]. All these facts indicate the need to develop new rapid and reliable diagnostic methods for brucellosis.

The purpose of this work was to develop a bacteriophage-based assay for highly sensitive and specific detection of live *Brucella* in liquid cultures and in blood, and potentially in other key biological samples. Before the development of a wide array of automated systems for blood culture identification and PCR-based molecular tests, typing with a panel of diagnostic brucellaphages was the tool of choice for species determination of *Brucella* spp. [27,32,33]. The use of phages for this purpose is still included in the Manual of Diagnostic Tests and Vaccines for Terrestrial Animals [40] and is applied globally as a complementary method [41,42,43,44]. The intrinsic ability of phage to specifically infect and amplify in live host bacteria is a feature very important for phage-based diagnostics. Standard phage amplification assays based on plaque formation (also called phage titer increase tests) have been used since the early 1960s for the detection of *Shigella*, *Salmonella*, *Listeria* and other pathogenic bacteria [45,46]. These assays remain important for the detection of slow growing bacteria, such as *Mycobacterium tuberculosis* [47,48] and *Mycobacterium paratuberculosis* [49,50]. Phage amplification assays have been improved using several quantitative approaches for phage detection, including high-performance liquid chromatography [51], qPCR [34,35,52,53], competitive ELISA [54], and lateral flow immunoassay [55]. Another group of methods measure the extent of phage-based bacterial lysis using fluorescent or amperometric detection of released products, like adenylate kinase [56] and β-galactosidase [57]. Finally, several genetically engineered reporter phages have been successfully used for indirect fluorescent detection of *M*. *tuberculosis* [58,59], *Y*. *pestis* [60] and *B*. *anthracis* [61].

We demonstrated previously that one particle of a plague diagnostic phage φA1122 is reproduced in 57 copies (burst size) upon its propagation in one *Y*. *pestis* cell during 30 min [34]. An assay for qPCR monitoring of amplification of this specific phage in the presence of *Y*. *pestis* was developed that is rapid (4 h), highly sensitive (one CFU per sample) and specific for indirect detection of the plague bacterium, and which performed well with various clinical samples [34,35]. Since phages propagate only on viable and culturable cells [62], the method allows the identification of only live bacteria that gives some additional important clinically and epidemiologically relevant information in comparison with assays targeting bacterial DNA. Another advantage of indirect phage-based detection is that there is no need in phage DNA extraction and purification [34,35]. qPCR targeting phage γ has been employed for rapid and sensitive indirect detection of *Bacillus anthracis*, though assay specificity was not tested using near-relative *Bacillus cereus* [53].

It was previously shown that lysis of *B*. *abortus* by phage Tb is delayed but very robust, producing about 121 phage particles from one bacterium [36]. In the present study we employed brucellaphage amplification monitored by qPCR for indirect detection of *B*. *abortus*. Five brucellaphages from the typing panel, Tb, S708, Fz, Wb, and Bk (see Table 2), were studied as potential reporters. Testing quantitative parameters of qPCR with purified DNA of phages Tb, S708, Wb, and Bk showed reliable standard curves down to 0.35–0.5 fg (10–15 genome equivalents; as an example, see Figure 1a); based on Tb burst size this is equivalent to 0.05–0.1 cells of *B*. *abortus*. Conducting the qPCR with dilutions of phage lysates (without DNA purification) yielded similar curves (Figure 1b). However, calculated phage genome equivalents were somewhat higher than the numbers of live phage particles. For example, 1 µL of 10^−6^ dilution of S708 (with initial concentration of 2 × 10^9^ PFU/mL) is expected to contain only two live phage particles, whereas qPCR data showed a signal equivalent to 641 phage genomes (Figure 1b). This discrepancy can be explained by the previous observation that the treatment of brucellaphage lysates with 10% chloroform decreases the number of live phage [36]. For this reason the authors [36] proposed that brucellaphage lysates be treated with toluene instead of chloroform. However, since this discrepancy did not impair assay efficiency, for the purposes of this study chloroform was used in all experiments to standardize the procedure with previously developed assays [34,35] and to reduce the risk of laboratory exposure to live *B*. *abortus* S19, which is known to cause human infection [63].

The indirect phage-based *Y*. *pestis* detection assay developed previously in our laboratory allowed the detection of a single bacterium per sample in 4 h [34,35]. However, in the case of *B*. *abortus* and S708 there was no phage burst detected by qPCR after 6 or 8 h of incubation (Figure 2). The initial burst was observed only at the highest concentrations of *B*. *abortus* (10^8^–10^6^) and after 18 to 24 h of phage propagation. However, the increase in phage titer in this burst was remarkable, about five to six orders of magnitude above the starting titer. After 48 h the phage burst was noted at lower bacterial concentrations, 10^5^–10^3^ CFU/mL, and after 72-h incubation the marked burst was observed at the lowest concentrations (10^2^–10^0^ CFU/mL). Remarkably, the final yield of bacteriophage after 72 h of incubation was almost the same at any given concentration of *Brucella* starting culture (Figure 2). Thus the lower limit of detection for indirect S708 phage-based qPCR-assisted detection of *B*. *abortus* was about one cell per mL of test culture, indicating the high sensitivity of the assay. Testing of four additional brucellaphages capable of propagating on *B*. *abortus* (Tb, Fz, Wb, and Bk) showed that all these phages can robustly and consistently amplify on low concentrations of *B*. *abortus* down to a single bacterium per milliliter of culture (the detection limit was the same, about 10^0^ CFU/mL) after 72 h (Figure 3), and thus can be efficient reporters in the indirect detection approach. Since brucellaphage Bk (both at high titer and diluted) has been shown to lyse smooth cultures of many *Brucella* spp. including *B*. *abortus*, *B*. *suis*, *B*. *melitensis*, *B*. *neotomae* [32,37], and *B*. *microti* [38], we therefore expect that this phage will be an efficient reporter for the indirect detection of the majority of *Brucella* strains that cause human brucellosis.

In order to test assay specificity, we used brucellaphage Bk and six bacterial species of different taxonomic groups (Table 2). Of them, *O*. *anthropi* is the nearest phylogenetic relative of *Brucella*, an agent of opportunistic infections and a frequent cause of misdiagnosis [64,65,66]. *Y*. *enterocolitica* serovar O:9 was found to have common antigens with *Brucella* that result in cross-reactivity when doing serologic tests for brucellosis and yersiniosis [67,68,69]. The genus *Afipia* also belongs to α-proteobacteria, order *Rhizobiales*, along with *Brucella* and *Ochrobactrum*, and it has been reported that *Afipia clevelandensis* has antigens that are cross-reactive with *Brucella* and *Y*. *enterocolitica* O:9 [70]. The lack of propagation of the Bk phage after 72 h incubation with all of the bacterial cultures tested other than the positive control indicates that the assay is specific for *B*. *abortus*. Moreover, the Bk phage was demonstrated to efficiently amplify on *B*. *abortus* in the mixed cultures containing faster growing *O*. *anthropi* or *Y*. *enterocolitica* O:9 (Figure 4).

The clinical relevance of the bacteriophage-based qPCR assay was evaluated first with sheep blood, plasma and serum artificially contaminated with *B*. *abortus*. We did not detect any Bk phage propagation in any of these blood products. Since all these products contain sheep serum, it appears there is a serum component (or components) that inhibits brucellaphage propagation. Thus a simple and short (10 min) lysis-centrifugation procedure was introduced to concentrate and wash *B*. *abortus* cells from sheep blood samples before subsequent Bk phage amplification. The processed samples then provided robust brucellaphage propagation within 72 h, with a lower limit of detection of approximately three *B*. *abortus* cells per milliliter of blood (Figure 5). The analytical sensitivity of previously described qPCR assays targeting different *Brucella* genes that used DNA purification methods depended upon the gene target and varied from 200 to 1 fg of DNA per reaction, equivalent to 4 × 10^4^ to 5 × 10^2^ CFU/mL [71,72,73,74,75,76]. In one report, one of six tested commercial DNA extraction kits allowed detection of 10^0^ CFU/mL of *B*. *abortus* in suspension [24]. Detection of *Brucella* by qPCR from whole blood [77] and serum [78] provided the detection levels of about 2 × 10^3^ and 7 × 10^3^ CFU/mL, respectively, and the best result obtained from serum samples was 5 CFU/mL [79]. Our method improves sensitivity because it allowed the consistent detection of single *B*. *abortus* cells per milliliter of both culture suspensions and blood.

Since phages amplify only on viable bacterial cells [62], our approach can be considered as an analog of *Brucella* culture methods, but more rapid and much more sensitive. While phage-based detection takes longer to perform than PCR assays targeting *Brucella* DNA, our method has improved sensitivity and adds information about the presence of live *Brucella* in clinical samples that is important for diagnosis, therapy and prognosis of brucellosis. The highly sensitive, specific and rapid method of indirect detection of *B*. *abortus* using qPCR monitoring of phage amplification that we developed proved effective with pure and mixed cultures and with blood spiked with *Brucella* cells. We expect this assay to be readily adaptable not only to blood samples from humans and livestock but also to other relevant biological samples, and expect this assay to detect not only *B*. *abortus* but also other pathogenic *Brucella* species.

## 4. Materials and Methods

### 4.1. Bacterial Strains, Bacteriophages and Growth Media

All bacteriophages used in this work were commercially acquired from Félix d’Hérelle Reference Center for Bacterial Viruses (Université Laval, Quebec City, QC, Canada). BBLBrucella Broth, BBLBrucella Agar, Tripticase Soy Broth, and Tripticase Soy Agar (BD Biosciences, San Jose, CA, USA) were used for bacterial and phage growth. Brucellaphages and bacterial strains used in this work are listed in Table 2.

Brucellaphages were propagated on attenuated animal vaccine strain *B*. *abortus* S19, as described earlier [31], with some modifications as follows. Phage stock lysate was added to 150 mL of early logarithmic phase liquid bacterial culture at OD_600_ of <0.1 and multiplicity of infection (MOI) of 0.1 and incubated for 48 h at 37 °C in a 1000 mL vented plastic Erlenmeyer flask with shaking at 60 rpm. After incubation, phage lysate was allowed to stand undisturbed at 4 °C for 24 h. The lysate was treated with chloroform at a final concentration of 10% for 1 h in order to destroy all remaining live bacteria. The bacterial debris was removed by centrifugation at 5000× *g* for 15 min, and the resulting lysate was filtered through 0.22 µm filter. Phage titers were determined by spotting 10-fold dilutions of lysates on semi-solid agar overlay containing *B*. *abortus* S19.

### 4.2. Phage DNA Isolation

Brucellaphage lysates were concentrated by centrifugation at 13,250× *g* for 3 h and resuspended in SM buffer (Teknova, Hollister, CA, USA), at 1/100 of the original volume. The concentrated lysate was incubated for 1 h at 37 °C with 60 µg/mL of RNAse A and 20 µg/mL of DNAse I to remove *Brucella* DNA and RNA. Then phage suspension was treated with proteinase K (50 µg/mL) and sodium dodecyl sulfate (SDS; 0.5%) for 1 h at 56 °C. DNA was extracted by phenol:chloroform method, ethanol precipitated [87] and resuspended in nuclease-free water. DNA purity was confirmed by restriction analysis. DNA concentration was determined on a Nanodrop 2000 machine (Thermo Scientific, Wilmington, DE, USA).

### 4.3. Phage S708 Amplification on Different Culture Dilutions of *B. abortus*

An overnight culture of *B*. *abortus* S19 was diluted to OD_600_ = 0.1 in Brucella Broth. 20 mL of each serial bacterial dilution from 10^0^ to 10^−8^ (ca. 10^8^ to 10^0^ CFU/mL, respectively) were made in Brucella Broth and placed into conical Erlenmeyer flasks covered with ventilated aerosol-proof closure caps. The live bacteria counts were performed by plating 100 µL of 10^−6^ dilution on Brucella Agar in triplicate for each independent experiment. Diagnostic bacteriophage S708 was added to each flask to a final concentration of 10^3^ PFU/mL, and the sample was incubated in a shaker incubator at 37 °C and 60 rpm. 500 µL samples were withdrawn after 0, 6, 24, 48, and 72 h of incubation. Samples were treated with chloroform added to a final concentration of 10% and centrifuged in an Eppendorf mini-centrifuge for 3 min to remove *Brucella* cell debris. One microliter of each sample, neat or diluted 1:100 with Brucella Broth, was used for qPCR analysis (see below).

### 4.4. Testing Different Brucellaphages for qPCR Limits of Detection

Stock lysates of brucellaphages Tb, Fz, Wb, and Bk were diluted in SM buffer and added at 10^3^ PFU/mL to the culture of *B*. *abortus* S19 diluted in Brucella Broth at concentrations of ~1 × 10^2^; ~1 × 10^1^ and ~1 × 10^0^ CFU/mL. Bacterial titer was confirmed by the live bacteria count of cultures plated on Brucella Agar. qPCR was performed after 72 h of incubation of each test culture with corresponding bacteriophage.

### 4.5. Testing Phage Specificity on Non-*Brucella* Bacteria

Bacterial cultures used for testing phage specificity (*Escherichia coli*, *Yersinia enterocolitica* O:9, *Ochrobactrum anthropi*, *Afipia felis*, and *B*. *anthracis*, see Table 2) were grown overnight in Brucella Broth. Ten microliters of overnight culture were diluted with 10 mL of Brucella Broth (1:1000), and 10 µL of brucellaphage Bk (10^6^ PFU/mL) were added, to get a final concentration of 10^3^ PFU/mL. As a positive control, we used overnight culture of *B*. *abortus* S19 with the same concentration of Bk added. All cultures were incubated at 37 °C for 72 h with shaking at 200 rpm. The resulting culture was treated with 10% chloroform, a 0.5 mL aliquot was withdrawn and centrifuged for 3 min in a mini-centrifuge. One microliter of the resulting lysate was used for qPCR reaction. For testing phage specificity in mixed cultures, the equal volumes of diluted overnight non-*Brucella* and *B*. *abortus* S19 cultures were mixed. Samples were grown in the presence of the Bk phage at 10^3^ PFU/mL for 72 h, followed by chloroform treatment and centrifugation as described above.

### 4.6. Phage Amplification in Simulated Clinical Samples Containing *B. abortus*

Simulated clinical tests were performed in Na_2_-EDTA-treated whole sheep blood (LAMPIRE Biological Laboratories, Inc. Pipersville, PA, USA). Overnight culture of *B*. *abortus* S19 was diluted to OD_600_ = 0.1 in Brucella Broth. Then serial bacterial dilutions 10^−2^ (~3 × 10^6^ CFU/mL), 10^−4^ (~3 × 10^4^ CFU/mL), 10^−6^ (~3 × 10^2^ CFU/mL), 10^−7^ (~3 × 10^1^ CFU/mL), and 10^−8^ (~3 × 10^0^ CFU/mL) were made in sheep blood. The live bacteria count was done by plating 100 µL of 10^−6^ blood dilution on Brucella Agar. To optimize the indirect detection of *B*. *abortus* from blood, we used a lysis-centrifugation procedure [88] modified as follows. One milliliter of a blood sample was mixed with 0.5 mL of Red Blood Cell (RBC) Lysis Buffer (Sigma-Aldrich Co. LLC, St. Louis, MO, USA) containing 8.3 g/L of ammonium chloride and 0.01 M Tris-HCl, pH 7.5 ± 0.2. The resulting blood lysates containing intact leukocytes and *Brucella* cells were centrifuged for 4 min at full speed in a tabletop mini-centrifuge (Eppendorf North America, Hauppauge, NY, USA). The pellets were resuspended again in 1 mL of RBC Lysis Buffer to assure the complete removal of RBC and hemoglobin. After the second round of centrifugation, the pellets were resuspended in 1 mL of sterile water to break leukocytes by osmotic shock in order to simulate the release of intracellular Brucellae. Repeated determination of live *Brucella* was performed by plating 100 µL of 10^−6^ dilution of the washed blood sample. All live bacteria counts were done in three independent experiments in triplicate. The entire washed blood sample was inoculated into 20 mL of Brucella Broth in 125 mL Erlenmeyer flask with 0.22-µm filter cap, and bacteriophage was added at 10^3^ PFU/mL. The samples were incubated at 37 °C with shaking at 60 rpm for 72 h, then treated with chloroform, centrifuged and used as indicated above.

### 4.7. Primer Design

Primers for qPCR were designed by using the Beacon Designer program [89]. The target for brucellaphage-based assay was DNA primase/polymerase gene of phage S708, sequenced in our lab recently [30]. Primers (5′-CATACCAGATGGGTTGATAACTGTTGAG-3′ and 5′-ACTGTTTGTAAATAGACGCCAGAAG-3′) were analyzed using the BLAST (Basic Local Alignment Search Tool) engine at the National Center for Biotechnology Information (NCBI) web site against the nonredundant (nr) database [90], and were shown to be brucellaphage-specific, i.e., to have 100% identity only with DNA of brucellaphages sequenced to date: Tb, Pr [29], S708, Fz, Wb, Bk, R/C [30], and F1 [91]. The primers were also tested for specificity against *Brucella* genomic DNA (culture suspension of *Brucella abortus* S19 at about 5 × 10^8^ CFU/mL in distilled water, boiled for 5 min).

### 4.8. qPCR Assays

In all qPCR experiments, MaximaSYBR Green/ROX qPCR Master Mix 2× (Fermentas Inc., Glen Burnie, MD, USA) was used, according to the vendor’s recommendations. Reaction mixes were prepared in a total volume of 10 µL including 1 µL of DNA template (purified DNA solution or suspension containing live phage particles), 5 µL of the master mix, and 0.3 µM of each primer. Reactions were run on a LightCycler 2.0 (Roche Applied Science, Indianapolis, IN, USA). The cycling parameters were: 95 °C, 5 min; 40× (95 °C, 20 s; 60 °C, 60 s) with fluorescence measurement at the end of each cycle.

### 4.9. Statistical Analysis

Results of qPCR experiments are shown as the mean values of at least three independent tests. Statistical significance was computed by One Way analysis of variance (ANOVA) free online program at the website of Vassar College [92]. *P*-values < 0.05 were considered significant.

## Figures and Tables

**Table 1:** Brucellaphage propagation: quantification by live counts and quantitative PCR (qPCR).

Bacteriophage	Titer After Propagation, PFU/mL *	Number of Genome Equivalents per mL **
Tb	2 × 10^10^	2.8 × 10^12^
S708	2 × 10^9^	6 × 10^11^
Fz	1 × 10^11^	2.5 × 10^12^
Wb	1 × 10^10^	1 × 10^12^
Bk	1 × 10^8^	2 × 10^12^

* Live phage counts were determined by spotting phage lysate dilutions on semi-solid agar overlays containing *Brucella abortus* S19; ** Concentration of phage genomes was calculated using DNA-based calibration curve established by qPCR; PFU: plaque-forming units.

**Table 2:** Bacteriophages and bacterial strains used in this work.

Bacteriophage or Bacterial Strain	Source	Reference
Brucellaphages:		
Tb (Tbilisi)	d’Hérelle Phage Collection *	[29,30,80]
S708	d’Hérelle Phage Collection *	[30,81]
Fz (Firenze)	d’Hérelle Phage Collection *	[30,81]
Wb (Weybridge)	d’Hérelle Phage Collection *	[30,81]
Bk (Berkeley)	d’Hérelle Phage Collection *	[30,37]
Bacterial strains:		
*Brucella abortus* S19	Laboratory collection	[82]
*Ochrobactrum anthropi* ATCC 49187	ATCC**	[83]
*Afipia felis* ATCC 53690	ATCC	[84]
*Bacillus anthracis* Sterne	Laboratory collection	[85]
*Escherichia coli* C600	Laboratory collection	[86]
*Yersinia enterocolitica* 2516-87 (O:9)	Laboratory collection	[34]

* Félix d’Hérelle Reference Center for Bacterial Viruses (Université Laval, Canada). **American Type Culture Collection, Manassas, VA, USA.
